# Novel Gene Signatures Promote Epithelial-Mesenchymal Transition (EMT) in Glucose Deprivation-Based Microenvironment to Predict Recurrence‐Free Survival in Hepatocellular Carcinoma

**DOI:** 10.1155/2023/6114976

**Published:** 2023-02-21

**Authors:** Yuan Huang, Shi-Rong Li, Ying-Jie Gao, Yan-hua Zhu, Xiao-feng Zhang

**Affiliations:** ^1^Department of Biochemistry and Molecular Biology, School of Bioscience and Technology, Chengdu Medical College, Chengdu, Sichuan, China; ^2^Laboratory of Animal Tumor Models, Frontiers Science Center for Disease-Related Molecular Network, State Key Laboratory of Biotherapy and Cancer Center, National Clinical Research Center for Geriatrics, West China Hospital, Sichuan University, Chengdu 610041, Sichuan, China

## Abstract

**Background:**

Current research studies have suggested that glucose deprivation (GD)-based tumor microenvironment (TME) can promote epithelial-mesenchymal transition (EMT) of tumor cells, leading to tumor invasion and metastasis. However, no one has yet studied detailedly the synthetic studies that include GD features in TME with EMT status. In our research, we comprehensively developed and validated a robust signature regarding GD and EMT status to provide prognostic value for patients with liver cancer.

**Methods:**

GD and EMT status were estimated with transcriptomic profiles based on WGCNA and t-SNE algorithms. Two cohorts of training (TCGA_LIHC) and validation (GSE76427) datasets were analyzed with the Cox regression and logistic regression analyses. We identified a 2-mRNA signature to establish a GD-EMT-based gene risk model for the prediction of HCC relapse.

**Results:**

Patients with significant GD-EMT status were divided into two subgroups: GD^low^/EMT^low^ and GD^high^/EMT^high^, with the latter having significantly worse recurrence-free survival (*P* < 0.01). We employed the least absolute shrinkage and selection operator (LASSO) technique as a method for HNF4A and SLC2A4 filtering and constructing a risk score for risk stratification. In the multivariate analysis, this risk score predicted recurrence-free survival (RFS) in both the discovery and validation cohorts and remained valid in patients stratified by TNM stage and age at diagnosis. The nomogram that combines risk score and TNM stage as well as age produces improved performance and net benefits in the analysis of calibration and decision curves in training and validation groups.

**Conclusions:**

The GD-EMT-based signature predictive model may provide a prognosis classifier for HCC patients with a high risk of postoperative recurrence to decrease the relapse rate.

## 1. Introduction

Hepatocellular carcinoma (HCC) accounts for 75–85% of all primary liver malignancies [1]. During the last decades, attempts at therapeutic approaches for HCC have been in progress not only for early stages but also for advanced stages, such as aggressive surgery, liver transplantation, chemotherapy with sorafenib, and multikinase inhibitors, all of which are recognized as effective treatments for sufferers [2, 3], as well as CAR-T cell dysfunction, which is considered a novel approach to affect the immune microenvironment and the immunotherapeutic response in HCC [4]. However, without targeted therapy, patients with early and advanced HCC have a poor prognosis, with a median survival of 6–9 months and 1-2 months, respectively [5]. Furthermore, a relapsed rate of 50%–70% has been achieved even after surgical resection of the lesion, let alone many patients who are not eligible for resection [6, 7]. Hence, the identification of novel panels providing more predictive value for sufferers' recurrence status is highly demanded clinically for improving the prognostication for liver cancer. Therefore, to improve the prognosis of liver cancer patients, there is a great clinical need to discover novel panels that have more predictive value for patients' recurrence status.

On account of the coevolution of malignant cells and their direct environment, the tumor forms an organ-like structure. Studies clearly show that cancer development and metastasis rely on the mutual cointeraction between tumor cells and their environment, which leads to the formation of the tumor microenvironment [8]. The rapid proliferation of cancer cells makes it often necessary for tumors to experience rapid angiogenesis, hypoxia, acidosis, glucose deprivation, immune cell infiltration, and decreased activity, which all contribute to the development of cancer as well as drug resistance [9, 10]. Thus, TME is equipped with low pH values, glucose deprivation (GD), severe hypoxia, high glutathione (GSH) content, and excessive hydrogen peroxide (H_2_O_2_) [11]. Accumulated evidence has testified that tumor cells have high migratory potential for constantly situating in glucose deprivation-based TME [12–14]. The epithelial-mesenchymal transition (EMT) refers to the transition from polarized epithelial cells to motile mesenchymal cells through the activation of a series of signals that enhance tumor stem cell-like properties, invasion, and metastasis [15]. Current research has suggested that a GD-based microenvironment can promote EMT of tumor cells, leading to tumor invasion and metastasis [16, 17]. Although there is an explicit link between the GD status and the EMT phenomenon of TME, an integrated analysis of the relationship between the GD state and the EMT response is rare.

In this study, we synthetically developed and validated robust signatures of GD and EMT status to provide prognostic value for HCC patients. Firstly, we filtered GD-related differentially expressed genes (DEGs) and EMT-related DEGs associated with prognosis and applied them to model construction in silico. Furthermore, multiple independent HCC datasets were integrated to develop a risk score based on GD-EMT status, and the functional studies of relevant genes were validated in vitro. Ultimately, an original model incorporating GD-EMT status and clinicopathological features was indicated through a range of systematic analyses aimed at predicting RFS in liver cancer with universal applicability in clinical practice.

## 2. Materials and Methods

### 2.1. Data Retrieval and Preprocessing

**Figure 1:**
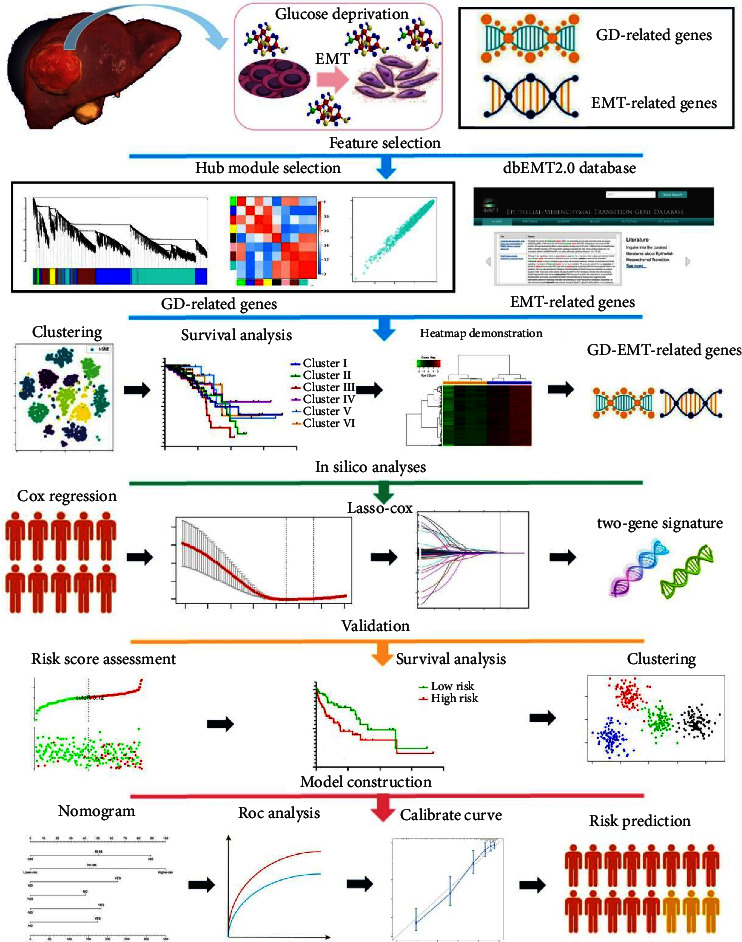
Schematic diagram of the study design. Feature selection: a panel of integrated GD-EMT-related DEGs was identified based on WGCNA and t-SNE algorithms; silico analyses: a two-gene signature was finally estimated using the LASSO-Cox regression models; validation: the crucial roles of novel signatures in GD and EMT status were further validated in multiple cohorts; model construction: nomogram establishment to predict RFS for HCC sufferers. GD, glucose deprivation; EMT, epithelial-mesenchymal transition; DEGs, differentially expressed genes; WGCNA, weighted gene coexpression network analysis; t-SNE, t-distributed stochastic neighbor embedding; LASSO, least absolute shrinkage and selection operator; RFS, recurrence-free survival; HCC, hepatocellular carcinoma.

The mRNA expression data and corresponding clinical characteristics of HCC patients were collected from The Cancer Genome Atlas (TCGA) cohort (https://portal.gdc.cancer.gov/) and the Gene Expression Omnibus (GEO) (https://www.ncbi.nlm.nih.gov/geo/). The study contained 418 HCC tumor samples with integrated clinical characteristics and valid survival data in TCGA database, 108 liver cancer patients from GSE76427 dataset, and 8 HepG2 cells treated with variously concentrated glucose (4 high glucose-relevant and 4 GD-related cells) from GSE140867 dataset. The mRNA expression matrix (FPKM) value from TCGA and GEO database was converted into TPM value. Besides, samples from TCGA cohort were randomly assigned to two phases, namely, training and internal validation cohort. The discovery set retrieved from the GSE76427 dataset was used for external validation. Thus, the tissue samples from TCGA-LIHC and GSE76427 datasets were assigned to diverse phases incorporating training, internal validation, entire, and external validation cohorts. Patients' clinicopathologic characteristics are listed in Table 1. All of the patients from the above two sets who met the following selection criteria could be enrolled: (a) histologically diagnosed malignant hepatocellular carcinoma; (b) eligible RNA expression; and (c) available RFS data. The workflow shown in Figure 1 was composed of feature selection, silico analysis, validation, and model construction.

### 2.2. Verification of GD Status and GD-Associative DEGs

We performed a weighted gene coexpression network analysis (WGCNA) using the WGCNA R package (version 3.613) to screen for genes associated with GD status and divided the associated mRNAs into the same coexpression modules [18]. On the basis of the results of the module-trait relationship, the module with the higher correlation was selected as the research object for the next study, and the genes in the pivotal module were considered as GD-related genes. Furthermore, t-distributed stochastic neighbor embedding (t-SNE) is a nonparametric and unsupervised algorithm that classifies or condenses patients into diverse clusters based on hub features or hallmarks by using the R package Seurat [19]. According to the RFS data, two clusters were singled out for comparison to determine the “GD^high^” and “GD^low^” groups. The limma algorithm was employed to filtrate DEGs between the two groups [20], and genes generated with a false discovery rate (FDR) corrected *P* value <0.05 and an absolute log_2_-fold change value >1 were regarded as GD-related DEGs.

### 2.3. Identification of EMT States and EMT-Related DEGs

There were 1184 EMT-related hallmark genes extracted from the dbEMT2.0 database (https://dbemt.bioinfo-minzhao.org/index.html), consisting of 1011 protein-coding genes and 173 noncoding RNAs. Similarly, patients were sectionalized into diverse clusters to compare the RFS data to ascertain the “EMT^high^” and “EMT^low^” groups. Thus, EMT-related DEGs could be further confirmed by the limma arithmetic, with the screening criteria of FDR-adjusted*P* < 0.05 and |log_2_FC| > 1.

### 2.4. Generation of GD-EMT Related DEGs

GD and EMT status identified above were divided into three groups, such as GD^low^/EMT^low^, GD^high^/EMT^high^, and mixed groups. The GD-EMT-related DEGs could be acquired by detecting expression differences between the GD^low^/EMT^low^ and GD^high^/EMT^high^ groups (FDR-adjusted*P* < 0.05, |log_2_FC| > 1). Finally, the set of genes most relevant to GD-EMT status can be obtained by comprehensively analyzing GD-EMT-associated DEGs and GD/EMT-associated DEGs distinguished above.

### 2.5. Establishment and Validation of Profiling Based on GD-EMT-Relevant DEGs

We performed univariate Cox regression analysis among GD-EMT-related DEGs using the R package “survival” and obtained preliminary GD-EMT-related DEGs that were significantly correlated with RFS in the training cohort, of which *P* < 0.05 was treated as significant. Afterwards, the least absolute shrinkage and selection operator (LASSO) method was used to calculate risk scores for HCC patients, which is characterized by preserving valuable variables and avoiding overfitting [21]. Based on these prognostic candidates, LASSO-Cox regression analysis was used to select genes to minimize the risk of overfitting. A risk prediction model was constructed and the penalty regularization parameter lambda (*λ*) was chosen through the cross-validation routine with an n-fold equal to 10 by using the R package glmnet. Meanwhile, lambda.min was identified to pick out the variables. Subsequently, we combined the regression coefficients from multivariate Cox regression models and optimized gene expression for each patient's risk score for RFS, that is, risk score = ∑coefficient (*i*) × expression of signature gene (*i*). The coefficients of gene (*i*) originated from the LASSO-Cox regression model, while its expression was derived from each patient. On the basis of risk scores, patients were divided into high-risk and low-risk groups. Besides, the Kaplan–Meier survival and principal component analysis (PCA) were drawn using the software “GraphPad prism 8.0” and the R package “rgl” for cluster analysis, respectively, to assess the predictive value of prognostic characteristics for RFS.

### 2.6. Enrichment Analysis

We conducted functional enrichment analysis using the package “cluster profiler” to explore potential molecules associated with GD-EMT-associated DEGs. Meanwhile, the correlations between risk scores and the enrichment scores of EMT-predicted pathways or GD-predicted pathways were conducted by the R package “ggcor.”

### 2.7. Construction and Assessment of the Nomogram

To estimate the feasibility of the risk score in depth, we selected patients with clinicopathological information from the TCGA dataset, which included age at diagnosis, alpha-fetoprotein (AFP) level, pathological tumor stage, gender, microvascular invasion, hepatitis B virus, and Barcelona Clinic Liver Cancer (BCLC) stage, and these characteristics were evaluated as categorical variables. Therefore, univariate and multivariate Cox regression analyses were performed to analyze the relationship between each variable and patient RFS. Nomograms are widely used for cancer prognosis, primarily because of their ability to reduce statistical predictive models into a single numerical estimate of the probability of an event, such as death or recurrence, which is tailored to the profile of an individual patient. The “rms” R package was utilized to construct nomograms. For 1-, 3-, and 5-year survival rates, calibration curves were used to quantify the agreement between the predicted and actual results. The ROC curve was developed with the R package “pROC” to evaluate the nomogram's efficiency.

### 2.8. Cell Culture and Staining

The human liver cancer cell line SMMC-7721 was obtained from the School of Bioscience and Technology, Chengdu Medical College (Chengdu, China). SMMC-7721 cells were cultured in high glucose (4500 mg/L) DMEM (Gibco) and low glucose (1000 mg/L) DMEM (Gibco), respectively. Both were supplemented with 10% fetal bovine serum (FBS) and 1% penicillin-streptomycin. Cells were then placed in a sterile incubator with 5% CO_2_ at 37°C. In addition, the cells were dyed with crystal violet after treatment with paraformaldehyde.

### 2.9. Wound-Healing Assay

The cells were cultured and when the cells were growing to about 90% confluence, scratched lines were evenly drawn on the bottom of the 6-well plate with a 20 *μ*l pipette tip and the dropped cells were gently washed with PBS. Complete medium containing 0.5% FBS was added to each well to continue the culture, and the healing of the scratches in the three fixed areas was photographed at 0 and 48 hours, respectively.

### 2.10. Cell Invasion and Migration Assays

Invasion assays were performed in 24-well Transwells (8 *μ*m pore size; BD), self-coated with Matrigel (356234; BD). Cells were added to a coated filter (5 × 10^4^ cells/filter) in 200 *μ*l of serum-free medium in triplicate wells. Next, 500 *μ*l of medium with 10% FBS was appended in the lower chamber. After 36 h, the upper surface of the filter was wiped off with a cotton swab. Cells on the lower surface of the membrane were fixed with 4% paraformaldehyde, stained with 0.5% crystal violet, photographed, and counted under a microscope in three random fields. Similarly, the migration assays were implemented with the same procedures, except that the plates were not coated with Matrigel and the plates were incubated for 12 h.

### 2.11. Statistical Analysis

The *R* version 3.6.1 (https://www.r-project.org) and the corresponding package were utilized for full data analysis. Cell experiments were repeated at least three times, and data was expressed using the mean ± standard error of the mean (SEM). Statistical analysis was achieved with a one-way ANOVA test using GraphPad Prism 8. Recurrence-free survival analysis was estimated using the Kaplan–Meier method. The value of *P* < 0.05 was considered statistically significant.

## 3. Results

### 3.1. EMT Occurrence in Glucose Deprivation-Based Microenvironment

**Figure 2:**
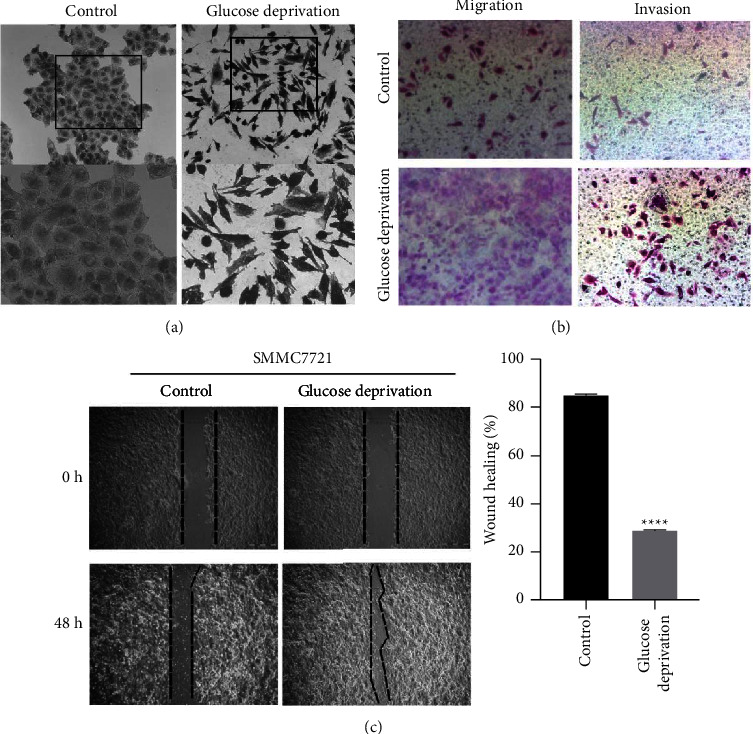
Glucose deprivation induces the EMT in HCC cells. HCC cells were exposed to glucose deprivation for 48 h. (a) Morphology of SMMC7721 cells in GD-based microenvironment. (b) Migration and invasion abilities were detected with glucose deprivation. (c) Scratch wound healing assay were used to evaluate the migration of SMMC7721 cell line in GD-based microenvironment. GD, glucose deprivation; EMT, epithelial-mesenchymal transition. ^*∗∗∗∗*^*P* < 0.0001.

EMT has been revealed to play an extremely significant role in the development and metastasis of tumors [22]. Previous studies have found that glucose deprivation treatment of tumor cells can lead to EMT induction and malignant transformation. An alteration of SMMC7721 cell lines from EMT-like phenotypes was evident after exposure to glucose deprivation for 48 hours (Figure 2(a)). Migration and invasion ability of SMMC7721 cells were significantly promoted by glucose deprivation (Figure 2(b)). Furthermore, cell migration and invasion ability were detected via the wound-scratch assay, the results of which showed that low glucose promoted the scratch healing ability of SMMC7721 cells (Figure 2(c)). In brief, our results showed that exposure to low glucose could induce EMT in HCC cells, which may participate in the malignant conversion.

### 3.2. Determination of GD Status and GD-Related DEGs in Liver Cancer

**Figure 3:**
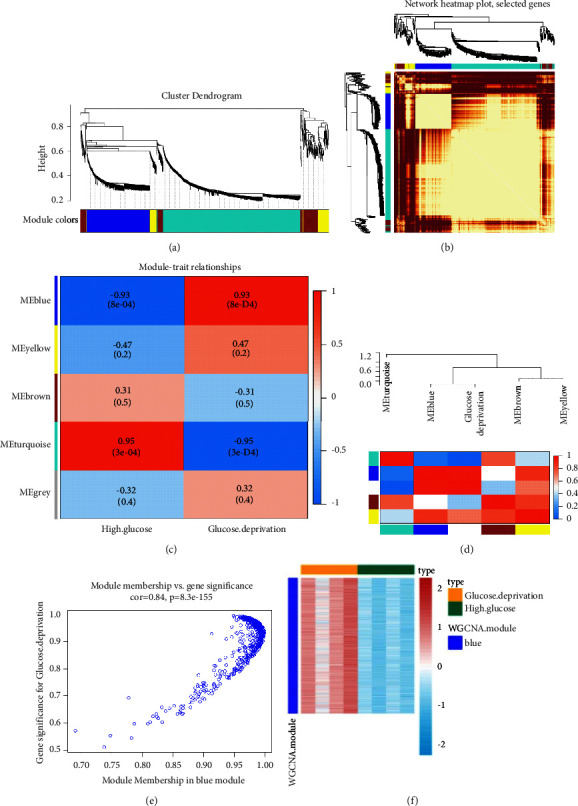
Coexpression network establishment to identify modules related with glucose deprivation based on WGCNA. (a) A clustering dendrogram of coexpression modules screened on the basis of glucose deprivation samples from GSE140867. (b) The heatmap analysis of the gene coexpression network. (c) Module-trait relationships between gene modules and glucose deprivation. (d) The blue module was significantly related with glucose deprivation. (e) The correlation between module membership and gene significance in blue module. (f) WGCNA clustering of differentially expressed genes in blue module. WGCNA, weighted gene coexpression network analysis.

**Figure 4:**
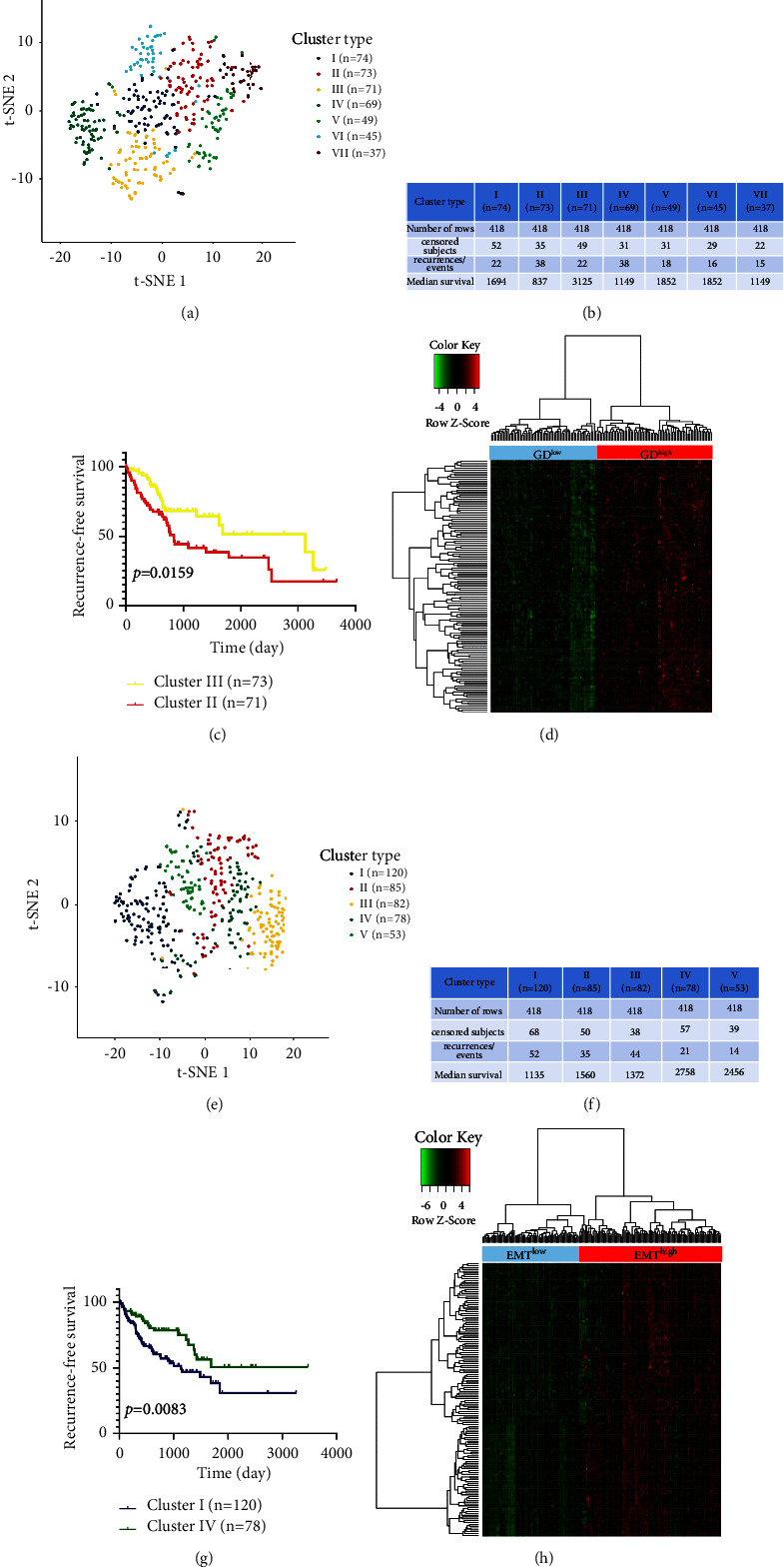
Identification of GD and EMT status and GD- and EMT-related DEGs. (a) Dot plot for seven distinct clusters identified by t-SNE algorithm based on 577 GD hallmark genes from blue module. (b) Recurrence-free survival analysis for seven clusters classified from 418 HCC patients. (c) Kaplan–Meier plot of RFS for HCC patients in Cluster II and Cluster III with worst- and best-prognosis. (d) Heatmap showing expression profiles for GD-related DEGs with comparison between GD^high^ and GD^low^ groups. (e) Five clusters identification by t-SNE algorithm on the foundation of 1011 EMT hallmark genes. (f) RFS comparison for five clusters generated from 418 sufferers. (g) HCC patients in Cluster I and Cluster IV yield worst and best RFS demonstrated by Kaplan–Meier plot. (h) EMT-related DEGs resulting from the comparison between EMT^high^ and EMT^low^ groups, revealed by heatmap. GD, glucose deprivation; EMT, epithelial-mesenchymal transition; RFS, recurrence-free survival.

We analyzed microarray datasets (GSE140867) generated from 4 high glucose-relevant and 4 GD-related HepG2 cells using WGCNA as a way to study hub modules in GD-positive samples, and finally screened 2269 genes with *P* < 0.05 and log_2_ (fold change)| > 1. After constructing the coexpression matrix, four gene modules, including blue model, yellow model, brown model, and turquoise model, were obtained by the dynamic hybrid shearing method (Figure 3(a)). Pearson correlation coefficients between expression profiles of all gene pairs were transformed into network connection strengths (indicated by intensity in red) (Figure 3(b)). After that, the heatmap exhibited the relevance between four gene modules and glucose deprivation, resulting in the correlation between the blue module and glucose deprivation achieving 0.93 (*P*=8*e* − 04) (Figures 3(c) and 3(d)). In addition, the correlation between module membership and gene significance was also analyzed, indicating that the blue module (*n* = 577 genes) may be especially critical for GD status (*r* = 0.84, *P*=8.3*e* − 155) (Figure 3(e)). Meanwhile, the WGCNA heatmap revealed that the gene expression profile in the blue module was significantly overexpressed in HCC cells with GD treatment, compared with HCC cells disposed by high glucose (Figure 3(f)). 418 liver cancer patients, considered as the discovery cohort, were derived from the TCGA database. The expression matrix of 577 GD hallmark genes in the blue module was adopted to compute the euclidean distance between any two individuals in the discovery cohort, and the nonlinear dimensionality reduction algorithm t-SNE was further applied to condense the euclidean distance into two-dimensional points. Subsequently, seven clusters with HCC patients were produced and every patient was allocated to the closest cluster (Figure 4(a)), namely, 74, 73, 71, 69, 49, 45, and 37 patients in seven distinct clusters (from Cluster I to Cluster VII), respectively. The RFS comparison showed that the most significant differences were uncovered between Cluster II and Cluster III (Figure 4(b)). Thus, patients in Cluster III produced the best RFS, but patients in Cluster II had the worst prognosis (*P*=0.0159; Figure 4(c)), suggesting that Cluster III and Cluster II perhaps represent the lowest and highest status of GD. Accordingly, sufferers in Cluster II and Cluster III were sectionalized into “GD^high^” and “GD^low^” groups, separately. To engender GD-related DEGs, the expression profiles of GD^high^ and GD^low^ groups were compared, resulting in 133 GD-related DEGs being confirmed (Figure 4(d)).

### 3.3. Determination of EMT Status and EMT-Related DEGs in Liver Cancer

1011 EMT hub genes were acquired from the hallmark gene sets in the dbEMT2.0 database. Likewise, t-SNE was applied to cluster the 418 HCC patients according to the expression profile of 1011 hallmark genes. Five clusters (120 patients in Cluster I, 85 patients in Cluster II, 82 patients in Cluster III, 78 patients in Cluster IV, and 53 patients in Cluster V) were grouped to analyze the relapse status among them (Figure 4(e)), which proved that the sufferers in Cluster IV generated the best RFS, regarded as the EMT^low^ group, compared with the patients in Cluster I, treated as the EMT^high^ group (*P*=0.0083; Figures 4(f), and 4(g)). A total of 225 DEGs correlated with EMT status were distinguished by comparing the expression matrix of the EMT^low^ and EMT^high^ groups (Figure 4(h)).

### 3.4. GD-EMT-Related Prognostic DEGs in Liver Cancer

**Figure 5:**
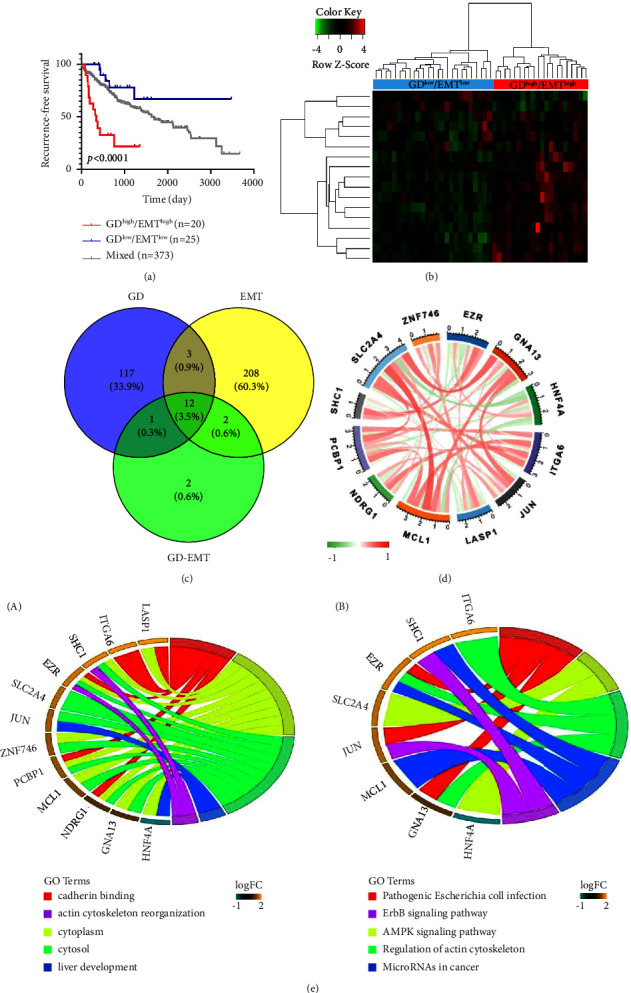
Identification and biological function of GD-EMT-related DEGs. (a) Kaplan–Meier plot of RFS for patients in three groups by uniting the GD and EMT status. (b) Heatmap displaying expression profiles for GD-EMT-related DEGs with comparison between GD^low^/EMT^low^ and GD^high^/EMT^high^ groups. (c) Venn diagrams show overlaps of GD-EMT-related DEGs with GD-related and EMT-related DEGs for discrimination of critical DEGs. (d) Correlation among the 12 GD-EMT-related DEGs. (e) Functional enrichment analysis, including GO enrichment analysis (A) and KEGG enrichment (B) analysis of the 12 GD-EMT-related DEGs. GD, glucose deprivation; EMT, epithelial-mesenchymal transition; GO, gene ontology; KEGG, Kyoto Encyclopedia of Genes and Genomes.

On the basis of the above results, a two-dimension index, combined with GD and EMT status, was further explored, that is, patients were divided into three sets: GD^low^/EMT^low^, GD^high^/EMT^high^, and mixed groups. RFS analysis revealed positive differences among the three groups (*P* < 0.001). Patients in the GD^low^/EMT^low^ group had the best RFS, while those in the GD^high^/EMT^high^ group had the worst RFS (Figure 5(a)), suggesting that GD and EMT have consistent clues to their effects on recurrence status in liver cancer patients. To obtain the DEGs associated with GD-EMT, expression profiles were compared between the GD^low^/EMT^low^ and GD^high^/EMT^high^ groups, drawing a conclusion on the identification of 17 GD-EMT-related DEGs (Figure 5(b)), including 2 overexpressed in GD^low^/EMT^low^ groups where patients had a higher survival rate and 15 overexpressed in GD^high^/EMT^high^ groups where patients had a poorer outcome. Consequently, 12 critical GD-EMT-related DEGs, generated from the overlap among 133 GD-related DEGs, 225 EMT-correlated DEGs, and 17 GD-EMT-related DEGs were filtered out (Figure 5(c)). Through Pearson correlation analysis, relevance among the 12 GD-EMT-related DEGs, such as SLC2A4, ZNF746, EZR, GNA13, HNF4A, ITGA6, JUN, LASP1, MCL1, NDRG1, PCBP1, and SHC1 was displayed (Figure 5(d)), among which the expression level of ZNF746, EZR, GNA13, HNF4A, ITGA6, LASP1, NDRG1, PCBP1, and SHC1 was decreased while the expression status of SLC2A4, JUN, and MCL1 was increased. Furthermore, enrichment analyses of functions revealed that the DEGs were associated with cadherin binding, cytoplasm, cytosal, liver development, and actin cytoskeleton reorganization in terms of gene oncology analysis. According to KEGG pathway analysis, the DEGs could take part in pathogenic *Escherichia coli* infection, the AMPK pathway, regulation of actin cytoskeleton, microRNAs in cancer, and the ErbB signaling pathway (Figure 5(e)).

### 3.5. Construction and Verification of a Comprehensive Index of GD-EMT-Based Gene Signature in Liver Cancer

**Figure 6:**
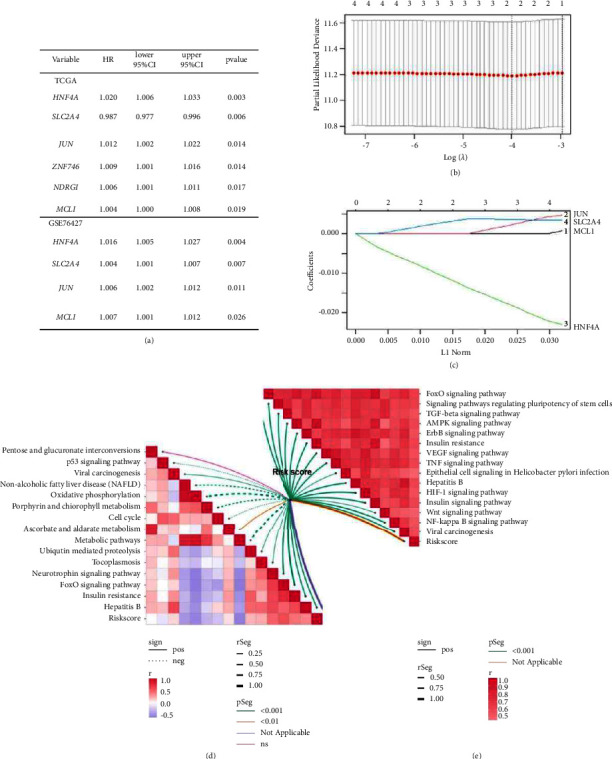
GD-EMT-based gene signature and prognosis classifier. (a) Univariate Cox regression analyses for GD-EMT-related prognostic DEGs in TCGA cohort and GSE76427 set. (b) LASSO coefficient distribution of 4 GD-EMT-related DEGs. (c) The tuning parameter (*λ*) in the LASSO model is chosen based on the minimum criterion (0.02036323). The correlations between risk score and the enrichment scores of GD-predicted pathways (d) as well as EMT-predicted pathways (e). GD, glucose deprivation; EMT, epithelial-mesenchymal transition; AUC, area under curve; DEGs, differentially expressed genes; LASSO, least absolute shrinkage and selection operator; HNF4A, hepatocyte nuclear factor 4 A; SLC2A4, solute carrier family-2-member-4-gene.

**Figure 7:**
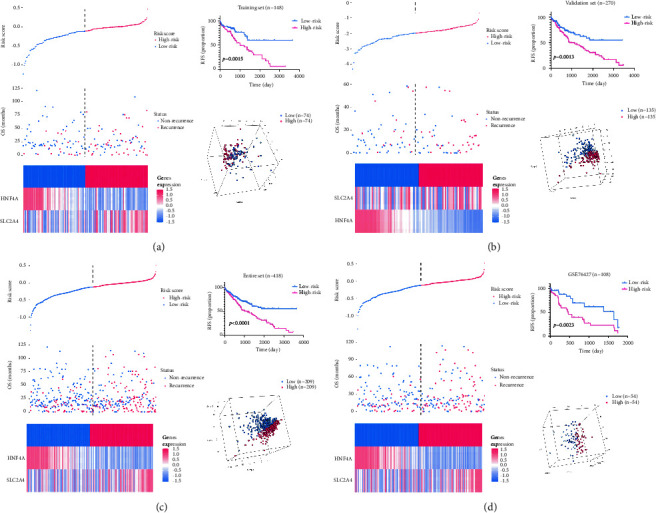
Construction of the two-gene signature to predict RFS for HCC patients. Assignment of risk score, recurrence status of the patients, the expression profiles of the two risk genes, Kaplan–Meier curves of RFS and PCA analysis according to the risk score, in the training cohort (a), validation cohort (b), entire cohort (c), and GSE76427 cohort (d), separately. RFS, recurrence-free survival.

In view of the EMT occurrence in GD-based microenvironment, a comprehensive analysis covering both GD and EMT status might emerge underlying prognostic value and quantify the TME. Thus, to distinguish GD-EMT-related prognostic DEGs, we further discriminated via univariate Cox regression overlapping 4 genes (SLC2A4, HNF4A, JUN, and MCL1) among the 12 DEGs in the TCGA cohort and GSE76427 set that have significant effects on patients' prognosis (*P* < 0.05; Figure 6(a)). To accomplish the establishment of a predictive model, the LASSO regression analysis was then performed to filter signatures from 4 GD-EMT-related prognostic DEGs. Cross-validation was also performed to obtain the best *λ* value from the smallest partial likelihood bias to further identify DEGs significantly associated with prognosis in liver patients. The corresponding coefficients were generated at the optimal log *λ* of 0.02036323. The results are shown in Figures 6(b) and 6(c). Thus, the risk score was calculated by the following formula: the combination mRNA panel = (−0.010913578 × expression value of HNF4A + 0.002874759 × expression value of SLC2A4). Meanwhile, the risk score was closely related to the expression of most enrichment scores of GD-predicted pathways (Figure 6(d)) and EMT-predicted pathways (Figure 6(e)). Subsequently, the patients were classified as high‐risk or low‐risk groups on account of the median value in training set (Figure 7(a)), validation set (Figure 7(b)), entire set (Figure 7(c)), and external testing set (Figure 7(d)). The number of relapsed patients increased with an increasing risk score. Consistently, SLC2A4 expression was upregulated along with the downregulated expression levels of HNF4A. The Kaplan–Meier analysis manifested that patients' RFS was longer in the low-risk group than that in the high-risk group (*P* < 0.01). Furthermore, PCA displayed that patients in diverse groups could be significantly clustered based on these two traits in all datasets. In conclusion, our findings indicated the applicability of the two-gene trait in recidivism prediction.

### 3.6. GD-EMT-Based Risk Score and Prognosis Classifier in Liver Cancer

**Figure 8:**
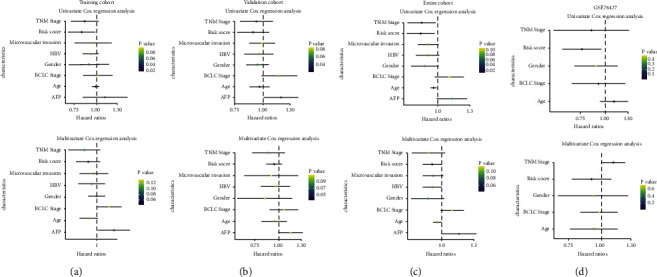
Univariate and multivariate Cox regression analyses of the risk score, patients' age, gender, HBV, tumor stage, microvascular invasion, AFP and BCLC stage in the training cohort (a), validation cohort (b), entire cohort (c), and GSE76427 cohort (d). The squares represent the hazard ratio (HR), and the black lines stand for the 95% CI. CI, confidence interval. Age, age at diagnosis; tumor stage, pathological tumor stage.

**Figure 9:**
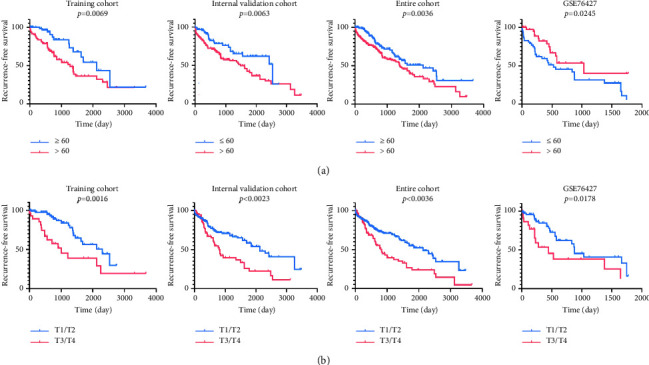
Kaplan–Meier curves of RFS according to patients' age at diagnosis (a) and clinical TNM stage (b) factors in the training cohort, validation cohort, entire cohort, and GSE76427 cohort. RFS, recurrence‐free survival.

**Figure 10:**
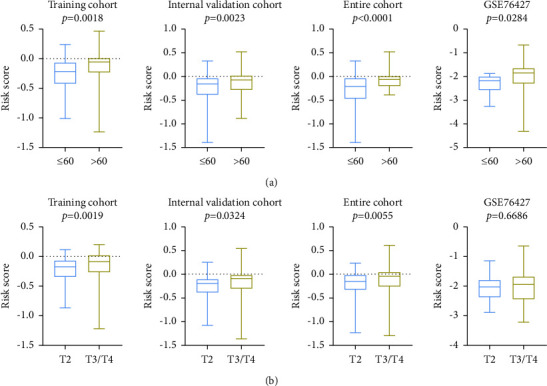
Associations of risk score with patients' age at diagnosis (a) and tumor stage (b) in the training cohort, validation cohort, entire cohort, and GSE76427 cohort, respectively.

**Figure 11:**
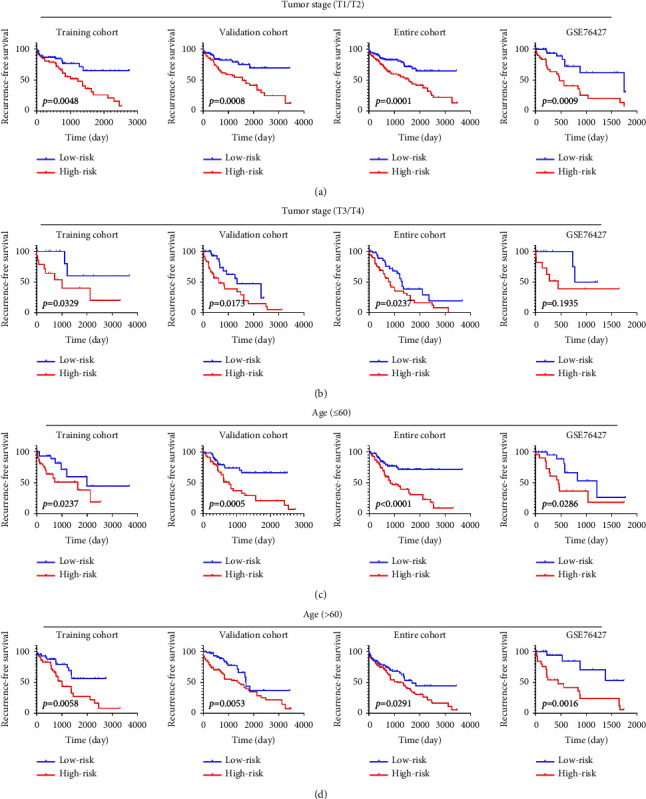
Kaplan–Meier curves of RFS according to the risk score in patients stratified by pathological tumor stage and patients' age at diagnosis. Kaplan–Meier curves were applied to patients with lower tumor stage (T1/T2) (a), higher tumor stage (T3/T4) (b), lower age at diagnosis (≤60) (c), and higher age at diagnosis (>60) (d) in the training cohort, validation cohort, entire cohort, and GSE76427 cohort, respectively. The tick marks on the Kaplan–Meier curves represent the censored subjects. The two‐sided log‐rank test was used to determine differences between the two curves. RFS, recurrence‐free survival.

The univariate analysis displayed that the risk score, age at diagnosis, and TNM stage were significantly associated with patients' RFS in the training cohort, validation cohort, the entire TCGA cohort, and external validation cohort with hazard ratios (HRs) of 0.828797988, 0.899926153, 0.867811632, and 0.774179934, respectively. Also, multivariate Cox regression analysis further demonstrated that the risk score remained as an independent prognostic factor after integrating with various clinicopathologic characteristics, including age at diagnosis, AFP levels, TNM stage, BCLC stage, HBV, gender, and microvascular invasion (Figures 8(a)–8(d), Table 2). Due to the significant relationship between age, TNM stage, and patients' prognosis, the prognostic values of a diversity of age and TNM stage were also explored. The Kaplan–Meier survival curves revealed that age and tumor stage could predict the outcome (Figure 9). As expected, a higher risk score was positively associated with older age (Figure 10(a)) and higher tumor stage in four cohorts (Figure 10(b)). The prognosis classifier was further validated within low-risk and high-risk patient subgroups with stage T1/T2 and stage T3/T4 in four cohorts, respectively. As a result, patients in the low-risk group were able to generate better RFS compared to the high-risk group in both the T1/T2 and T3/T4 stage subgroups (Figures 11(a) and 11(b)). Similarly, stratified analysis showed that risk scores could identify a different prognosis for sufferers with age ≤ 60 or older (>60) (Figures 11(c) and 11(d)).

### 3.7. Nomogram Based on Risk Score and Clinicopathological Features

**Figure 12:**
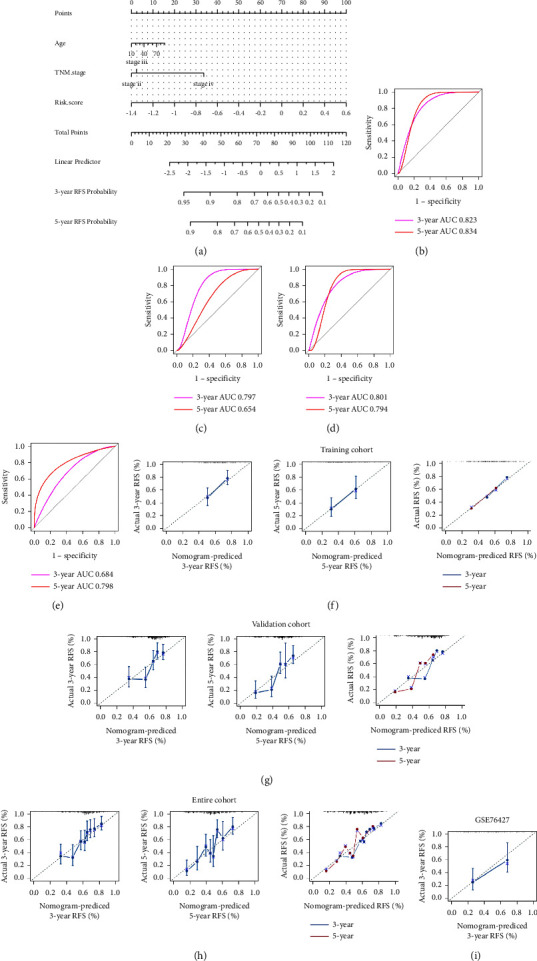
Nomogram construction and evaluation for predicting 3‐ and 5‐year RFS in HCC. (a) Nomogram establishment for the 3‐ and 5‐year RFS probability prediction in the TCGA training cohort. ROC curves evaluated the efficiency of the nomogram for predicting 3‐ and 5‐year RFS in the training cohort (b), internal validation cohort (c), entire cohort (d), and GSE76427 cohort (e). Calibration plots of the nomogram for predicting the probability of RFS at 3 and 5 years in the training cohort (f), internal validation cohort (g), entire cohort (h), and GSE76427 cohort (i). ROC, receiver operating characteristic.

We incorporated risk scores with clinicopathological characteristics (age at diagnosis and pathological tumor stage) to construct a nomogram to predict RFS. The points for each factor and total points were calculated separately to assess RFS rates at 3 and 5 years (Figure 12(a)), and then the validity of the nomogram was assessed using ROC curves and calibration plots, and the findings are shown in Figure 12(b). The 3- and 5-year AUCs for both the internal and external validation cohorts were smaller than those for the training cohort (0.797 and 0.654 and 0.684 and 0.798, respectively) (Figures 12(c) and 12(e)). The AUCs for the two time points were 0.801 and 0.794 in the TCGA cohort, respectively (Figure 12(d)). In addition, the calibration plots show excellent agreement between predicted and observed results in the internal validation cohort (Figure 12(g)), the training cohort (Figure 12(f)), the GSE76427 cohort (Figure 12(i)), and the TCGA cohort (Figure 12(h)).

## 4. Discussion

It is well known that the tumor microenvironment plays an important role in tumorigenesis by stimulating surrounding cells through the circulatory and lymphatic systems, which can further influence tumor development [23–25]. At the same time, it can reprogram the surrounding cells so that they play a decisive role in tumor survival. Malignant tumors with rapidly proliferating cells regularly experience nutrient (e.g., glucose) deprivation, which promotes tumor progression and aggressiveness through EMT induction [26]. Also, cells exposed to low glucose could suffer malignant transformation with elevated formation of colonies when compared to high glucose medium [27]. Through this study, we aimed to construct a model to solve the significant clinical issues by means of a comprehensive analysis of microenvironment characteristics and transcriptional profiles. Currently, the coincident effect of EMT status and GD is apparently related to recidivation after stratifying patients by clinicopathological risk factors. Finally, the GD-EMT-basedtwo-gene characteristics were used as prognostic classifiers for risk stratification and performed well in both the training and validation cohorts. Hence, this study synthetically analyzed the available HCC expression datasets to clarify GD-EMT-related DEGs to predict RFS for HCC sufferers. Besides, we systematically evaluated the prognostic value of risk scores in HCC patients to establish a model with better accuracy.

Previous studies have shown that the GD-based microenvironment drives the emergence of the EMT state in cancer, resulting in the invasion and metastasis of tumor cells [28–30]. However, few indicators regarding GD status have been developed, much less to focus on comprehensive effects between GD and EMT status, as well as their potential roles in clinically relevant classification. Moreover, determination of GD status by a single biomarker is not sufficient because it may be liable to omit important information about biological processes [31–34]. Thus, the implementation of combined GD-EMT features across cohorts can be used to develop continuous metrics for the comprehensive assessment of TME. The populations were divided into the GD^low^/EMT^low^ and GD^high^/EMT^high^ groups by subgroup classification, associated with different clinical prognosis, transcriptional GD-EMT patterns, and activation pathways that could be targeted for treatment. t-SNE has been used to discover potential subtypes of liver cancer, which provides an elegant dimensionality reduction technique [35–37]. In our study, t-SNE discerned disparate patterns of EMT status in TME based on a set of 1067 EMT hallmark genes from the dbEMT2.0 database, an updated database for EMT-related genes containing experimentally validated information and precomputed information on the regulation of cancer metastasis [38]. Also, EMT-predicted pathways during the EMT process were analyzed to explore their relationship with comprehensive features. When entering the GD state, GD-treated cancer cells without a specific genetic signature could be classified into diverse GD groups. Therefore, WGCNA, an effective method in many diseases that identifies modules of coexpressed genes [39], was employed to determine GD-related hub genes in one microarray dataset (GSE140867). As we all know, the invasive tumor cells in TME constantly exhibit dysregulated metabolism and enhanced aerobic glycolysis, leading to glucose depletion, hypoxia, immunosuppression, epigenetic modification, and lactic acid production [40, 41]. Nevertheless, the correlational studies were mostly concentrated upon the following aspects, such as hypoxia [42, 43], immune status [22, 44], RNA m6A methylation [45, 46], and lactic acid [47]. As a result, a synthetic study embracing GD characteristics in TME with EMT germination has not yet been studied in detail.

Studies have reported that the two signature genes in this study have a major role in multiple types of cancer. Hepatocyte nuclear factor 4 A (HNF4A), an orphan nuclear receptor, was one of the most important regulators of hepatocyte homeostasis, whose expression was frequently decreased in hepatocellular carcinoma. Cell invasion was closely associated with the downregulation of HNF4A expression, which promotes cancer metastasis [48, 49]. As for solute carrier family-2-member-4-gene (SLC2A4), encoding glucose transporter-4-protein (GLUT4), it has been reported to serve as a novel therapeutic candidate for cancer treatment [50]. The inhibition of SLC2A4 could compromise cell proliferation and metastasis in breast cancer [51], prostate cancer [52], and gastric cancer [53]. Thus, the two signature genes sifted from this study could offer latent candidates to elucidate molecular mechanisms in liver cancer.

There were a wide variety of potential targets with corresponding detailed mechanisms having been certified to be absolutely vital for tumor development. However, translating these efforts and discoveries from laboratory results to clinical applications is difficult. Hence, the incorporation of clinicopathological features and molecular markers could render a bran-new view for individualized treatment and prognostic observation. Our research provides a hint that patients in GDhigh/EMThigh status are considered high-risk patients, which can help clinicians make better decisions. In conclusion, this study linked microenvironmental characteristics and genetic profiles to patient prognosis, which could better serve the clinical therapeutics of patients with liver cancer.

There were a few limitations to this study. First, this study was performed by using bioinformatics analyses. Though we validated the results in several cohorts from public databases, we did not explore the relevant mechanisms in vivo experiments. Second, the clinical significance of the risk score needed further validation in prospective clinical trials. Third, we defined median cut-off values for risk scores in all cohorts rather than optimal cut-off values. Thus, findings in this study were waiting for further validation by well-designed, prospective, multicenter studies. Therefore, this study awaits further refinement with a well-designed multicenter prospective study.

## 5. Conclusion

In brief, the EMT changes due to the tumor GD microenvironment that were closely related to the prognosis of liver cancer patients. The GD-EMT-based genetic signature performed well in risk stratification and adds value beyond TNM staging. It can be used in clinical diagnosis for individualized treatment and prognosis, and follow-up can be scheduled on this basis.

## Figures and Tables

**Table 1:** Patient characteristics for the discovery and validation cohort.

Characteristics	Training set	Internal validation set	Entire set	External validation set	*P* value
No. of patients	148	270	418	108	
Age (y)					0.0059
<60 years	84	112	196	45	
≥60 years	64	158	222	63	
AFP (ng/ml)					0.2622
<4000	52	93	145	NA	
≥4000	96	177	273	NA	
Gender					0.5660
Female	33	54	87	93	
Male	115	216	331	15	
HBV					0.7793
Negative	10	18	28	NA	
Positive	138	252	390	NA	
TNM stage					0.0147
I	77	114	191	55	
II	27	67	94	35	
III/IV	44	89	133	18	
Microvascular invasion					0.0061
No	61	147	208	NA	
Yes	87	123	210	NA	
BCLC stage					0.6947
A	140	209	349	74	
B	8	60	68	23	
C	0	1	1	11	
Recurrence status					0.0275
Yes	59	114	173	44	
No	89	156	245	64	

**Table 2:** Univariate and multivariable analyses of risk score and clinicopathological characteristics for recurrence-free survival.

Study	Variables	Univariate analysis		Multivariable analysis	
		HR	*P* value	HR	*P* value
Entire	Risk score	0.867811632 (0.773751605–0.973305935)	**0.015**	0.923734351 (0.855578487–0.99731955)	**0.042**
	Age (≤60 vs. >60)	0.967260564 (0.940197009–0.995103142)	**0.021**	0.96876434 (0.932312491–1.006641395)	0.104
	TNM stage (T1/T2 vs. T3/T4)	0.881528852 (0.785152729–0.989734977)	**0.033**	0.89499416 (0.780079899–1.026836556)	0.113
	AFP (≤400 vs. >400)	1.123685871 (0.990186589–1.275183839)	0.071	1.151711447 (1.00295978–1.322524873)	0.085
	Gender (male vs. female)	0.903538025 (0.805220091–1.013860647)	0.084	0.890209034 (0.77623814–1.020913655)	0.096
	HBV (negative vs. positive)	0.921522173 (0.837464062–1.014017381)	0.094	0.925004708 (0.855938044–0.99964444)	0.099
	Microvascular invasion (no vs. yes)	0.928629563 (0.851249823–1.013043225)	0.095	0.927992472 (0.854632904–1.00764904)	0.095
	BCLC stage (A vs. B/C)	1.102550859 (0.978870018–1.241858851)	0.107	1.095101764 (0.995456697–1.20472128)	0.109

Training	Risk score	0.828797988 (0.694024082–0.989743906)	**0.018**	0.949534858 (0.87914574–1.025559707)	**0.044**
	Age (≤60 vs. >60)	0.988897728 (0.943559146–1.036414855)	**0.020**	0.951997381 (0.897249033–1.01008636)	0.120
	TNM stage (T1/T2 vs. T3/T4)	0.856672993 (0.720158203–1.019065829)	**0.031**	0.924170797 (0.840489422–1.01618371)	0.093
	AFP (≤400 vs. >400)	1.115476741 (0.839109974–1.482866845)	0.062	1.114063249 (1.00299592–1.237429682)	0.088
	Gender (male vs. female)	0.913834704 (0.703092164–1.187744522)	0.071	1.000764202 (0.943277472–1.06175438)	0.099
	HBV (negative vs. positive)	0.927290228 (0.841655711–1.021637654)	0.087	0.971989762 (0.893471997–1.05740762)	0.109
	Microvascular invasion (no vs. yes)	0.957709519 (0.757011709–1.211616033)	0.089	0.985033701 (0.904936057–1.07222094)	0.117
	BCLC stage (A vs. B/C)	1.019381469 (0.846122308–1.228118642)	0.099	1.092637219 (1.007298971–1.18520531)	0.132

Validation	Risk score	0.899926153 (0.763994426–1.060043181)	**0.021**	0.948309564 (0.856766115–1.04963422)	**0.035**
	Age (≤60 vs. >60)	0.953912331 (0.872148458–1.043341562)	**0.030**	0.955995187 (0.80898652–1.129718201)	0.097
	TNM stage (T1/T2 vs. T3/T4)	0.932652959 (0.790440597–1.100451503)	**0.041**	0.879600161 (0.713118416–1.08494806)	0.093
	AFP (≤400 vs. >400)	1.183162432 (0.992735979–1.410116457)	0.060	1.159666796 (0.998917703–1.34628416)	0.105
	Gender (male vs. female)	0.942625084 (0.841514345–1.055884612)	0.071	0.868183202 (0.618530124–1.21860204)	0.109
	HBV (negative vs. positive)	0.958316295 (0.83002329–1.10643898)	0.076	0.955995187 (0.80898652–1.129718201)	0.110
	Microvascular invasion (no vs. yes)	0.988063741 (0.870812937–1.121101807)	0.085	0.937039532 (0.66952759–1.311436746)	0.110
	BCLC stage (A vs. B/C)	1.17185982 (0.980610194–1.400409099)	0.081	1.062921205 (0.879592597–1.28445998)	0.108

GSE76427	Risk score	0.774179934 (0.614359762–0.975575888)	**0.010**	0.925763545 (0.768497885–1.1152121)	**0.027**
	Age (≤60 vs. >60)	1.124631434 (0.946611055–1.336130458)	**0.018**	1.117058604 (0.997657383–1.25074995)	0.045
	TNM stage (T1/T2 vs. T3/T4)	0.849454434 (0.544247537–1.325817365)	**0.027**	0.938078004 (0.754288539–1.16664949)	0.566
	Gender (male vs. female)	0.911922144 (0.705340644–1.179007622)	0.482	0.932012532 (0.679102757–1.27911034)	0.669
	BCLC stage (A vs. B/C)	0.932012532 (0.679102757–1.279110342)	0.043	0.969014711 (0.825409883–1.13760391)	0.701

^*∗*^Statistically significant with P-value <0.05.

## Data Availability

The research data used to support the findings of this study are available. The database can be obtained from the corresponding author upon request.

## Abbreviations

HCC: Hepatocellular carcinoma
TME: Tumor microenvironment
GSH: Glutathione
H_2_O_2_: Hydrogen peroxide
GD: Glucose deprivation
EMT: Epithelial-mesenchymal transition
DEGs: Differentially expressed genes
RFS: Recurrence-free survival
TCGA: The cancer genome atlas
GEO: Gene expression omnibus
WGCNA: Weighted gene coexpression network analysis
t-SNE: t-distributed stochastic neighbor embedding
FDR: False discovery rate
GMM: Gaussian finite mixture model
ROC: Receiver operating characteristic
AUCs: Area under curves
LASSO: Least absolute shrinkage and selection operator
PCA: Principal component analysis
AFP: Alpha fetoprotein
BCLC: Barcelona clinic liver cancer
FBS: Fetal bovine serum
SEM: Standard error of the mean
GS: Gene significance
MM: Module membership
HRs: Hazard ratios
HNF4A: Hepatocyte nuclear factor 4 A
AMPK: AMP-activated protein kinase
SLC2A4: Solute carrier family-2-member-4-gene
GLUT4: Glucose transporter-4-protein.
